# Neoadjuvant immunotherapy and neoadjuvant chemotherapy in resectable non-small cell lung cancer: A systematic review and single-arm meta-analysis

**DOI:** 10.3389/fonc.2022.901494

**Published:** 2022-09-21

**Authors:** He Wang, Tingting Liu, Jun Chen, Jun Dang

**Affiliations:** ^1^ Department of Radiation Oncology, The First Hospital of China Medical University, Shenyang, China; ^2^ Department of Radiation Oncology, Anshan Cancer Hospital, Anshan, China; ^3^ Department of Radiation Oncology, Shenyang Tenth People’s Hospital, Shenyang, China

**Keywords:** non-small cell lung cancer, neoadjuvant, immune checkpoint inhibitor, chemotherapy, pathological response, meta-analysis

## Abstract

**Background:**

It remains uncertain whether neoadjuvant immune checkpoint inhibitor (nICI) is superior to neoadjuvant chemotherapy (nCT) in resectable non-small cell lung cancer. In addition, there are outstanding questions for nICI such as the ideal treatment mode and predictors.

**Methods:**

PubMed, Embase, Cochrane Library, Web of Science, and scientific meetings were searched for eligible single-arm or multi-arm trials until 31 December 2021. The primary outcomes of interest were major pathological response (MPR) and pathological complete response (pCR). The random-effect model was used for statistical analysis.

**Results:**

Twenty-four trials of nICI (n = 1,043) and 29 trials of nCT (n = 2,337) were identified. nICI combination therapy was associated with higher MPR (63.2%, 95% CI: 54.2%–72.1%) and pCR (35.3%, 95% CI: 27.4%–43.3%) rates compared to nCT (16.2%, 95% CI: 7.5%–25.0%, P < 0.001 and 5.5%, 95% CI: 3.5%–7.5%, P < 0.001) and nICI monotherapy (23.3%, 95% CI: 12.7%–33.8%, P < 0.001, and 6.5%, 95% CI: 1.7%–11.2%, P < 0.001). As for safety, nICI monotherapy had the best tolerability; nICI combination showed a similar surgical resection rate and higher R0 resection rate compared to nCT. PD-1 inhibitor and high PD-L1 expression (≥1% or ≥50%) were correlated with higher MPR and pCR rates compared to PD-L1 inhibitor and PD-L1 expression <1%.

**Conclusions:**

nICI combination therapy is associated with higher MPR and pCR rates compared to nCT and nICI monotherapy. PD-1 inhibitor seems to be superior to PD-L1 inhibitor. PD-L1 status appears to be predictive of MPR and pCR for patients receiving nICI.

**Systematic Review Registration:**

https://www.crd.york.ac.uk/PROSPERO/display_record.php?RecordID=278661, CRD42021278661.

## Introduction

Non-small cell lung cancer (NSCLC) accounts for approximately 85% of all lung cancers (1). Surgical resection is still the main treatment mode for stage 1–2 and selected stage 3A NSCLC, but quite a few patients will have local recurrence and distant metastasis (2, 3). The addition of neoadjuvant chemotherapy (nCT) can improve the 5-year survival rate by only 5% (4).

Given the superior efficacy and manageable toxicity of immune checkpoint inhibitor (ICI) in patients with metastatic and unresectable locally advanced NSCLC, there is increasing interest in examining the role of ICI as a neoadjuvant treatment in patients with resectable NSCLC. Initial findings from a series of clinical trials have supported the safety and/or antitumor efficacy of neoadjuvant ICI (nICI) (5–28). Nevertheless, whether nICI is superior to nCT remains uncertain due to lack of randomized control trials (RCTs) with long-term outcomes. Moreover, there are still outstanding questions for nICI, such as the selection of nICI monotherapy or combination, the ideal predictive biomarkers, and the ideal timing and duration of ICI administration.

In light of these issues, we performed a systematic review and meta-analysis to assess the role of nICI and made a comparison with nCT. Due to that majority of trials of nICI did not report long-term survival results, we used major pathological response (MPR) and pathological complete response (pCR) as the primary outcomes of interest because they might be predictive of the overall survival (OS) for patients with resectable NSCLC (29, 30).

## Methods

This systematic review and meta-analysis was conducted according to the Preferred Reporting Items for Systematic Reviews and Meta-Analyses (PRISMA) guidelines (31) (**Supplementary File: Table S1**), and the protocol was registered in PROSPERO with registration number CRD42021278661.

### Literature search strategy

We systematically searched PubMed, Embase, Cochrane Library, and Web of Science for available studies published from 1 January 2000 until 31 December 2021, using the search terms “non-small cell lung cancer”, “neoadjuvant”, “chemotherapy”, and “immune checkpoint inhibitors” or “PD-1/PD-L1 inhibitors”. The detailed search strategy was provided in **Supplementary File: Table S2**. Abstracts of recent scientific meetings, including the American Society of Clinical Oncology (ASCO), European Society for Medical Oncology (ESMO), and World Conference on Lung Cancer (WCLC), were also inspected. The reference lists of relevant studies were checked for additional articles.

### Inclusion and exclusion criteria

The inclusion criteria were as follows: (1) single-arm or multi-arm trials examining nICI and/or nCT in resectable NSCLC; (2) reported at least one of the following outcomes: MPR (defined as less than 10% viable tumor cells in the resected specimen), pCR (defined as no viable tumor cells in the resected specimen), objective response rate (ORR, defined as the proportion of patients achieving a complete response or a partial response evaluated by RECIST criteria), grade ≥3 treatment-related adverse events (TRAEs), surgical resection rate, and the incidence of surgical complication; and (3) published in English. For multi-arm trials, only arms of nICI or nCT were included. If multiple articles covered the same study population, the one with the latest and most comprehensive data was selected.

### Data extraction

The following information was extracted independently by two authors (HW and SL): first author, publication year, trial design, region, follow-up time, sample size, tumor stage, interventions, MPR, pCR, ORR, grade ≥3 TRAEs, surgical resection rate, R0 resection rate, and surgical complication.

### Quality assessment

Risk of bias of individual trials was independently assessed by two authors (HW and SL). The Cochrane Risk of Bias Tool (32) was used to assess risk of bias of RCTs examining nICI vs. nCT. The trials were finally classified as low (all domains indicated as low risk), high (one or more domains indicated as high risk), and unclear risk of bias (more than three domains indicated as unclear risk).

### Statistical analysis

The primary outcomes of interest were rates of MPR and pCR. Rate of MPR or PCR refers to the ratio of patients achieving an MPR or PCR to all patients undergoing surgical resection. The second outcomes of interest included ORR, incidence of grade ≥3 TRAEs, surgical resection rate (the ratio of patients who underwent surgical resection to those who were planned to), R0 resection rate (the ratio of patients achieving a R0 resection to all patients undergoing surgical resection), and the incidence of surgical complication (operation-related complications occurring during the perioperative period). The random-effect model was performed for statistical analysis, using the software R (version 4.1.1, R Foundation for Statistical Computing) *via* the meta package. The inverse variance method was used to calculate pooled estimates of the outcomes and their 95% CIs. Differences between nICI and nCT were tested with the Z test. The heterogeneity among studies was estimated by the chi-square (χ^2^) and I-square (I^2^) tests with significance set at a P value of less than 0.10 or I^2^ greater than 50%. In addition, subgroup analyses in patients receiving nICI were performed according to clinical stage, histological type, type of nICI combination therapy, type of nICI monotherapy, and PD-L1 expression. The stability of the results was assessed by sensitivity analysis. The funnel plot, Begg’s test (33), and Egger’s linear regression test (34) were performed to investigate publication bias.

## Results

### Eligible studies

**Figure 1:**
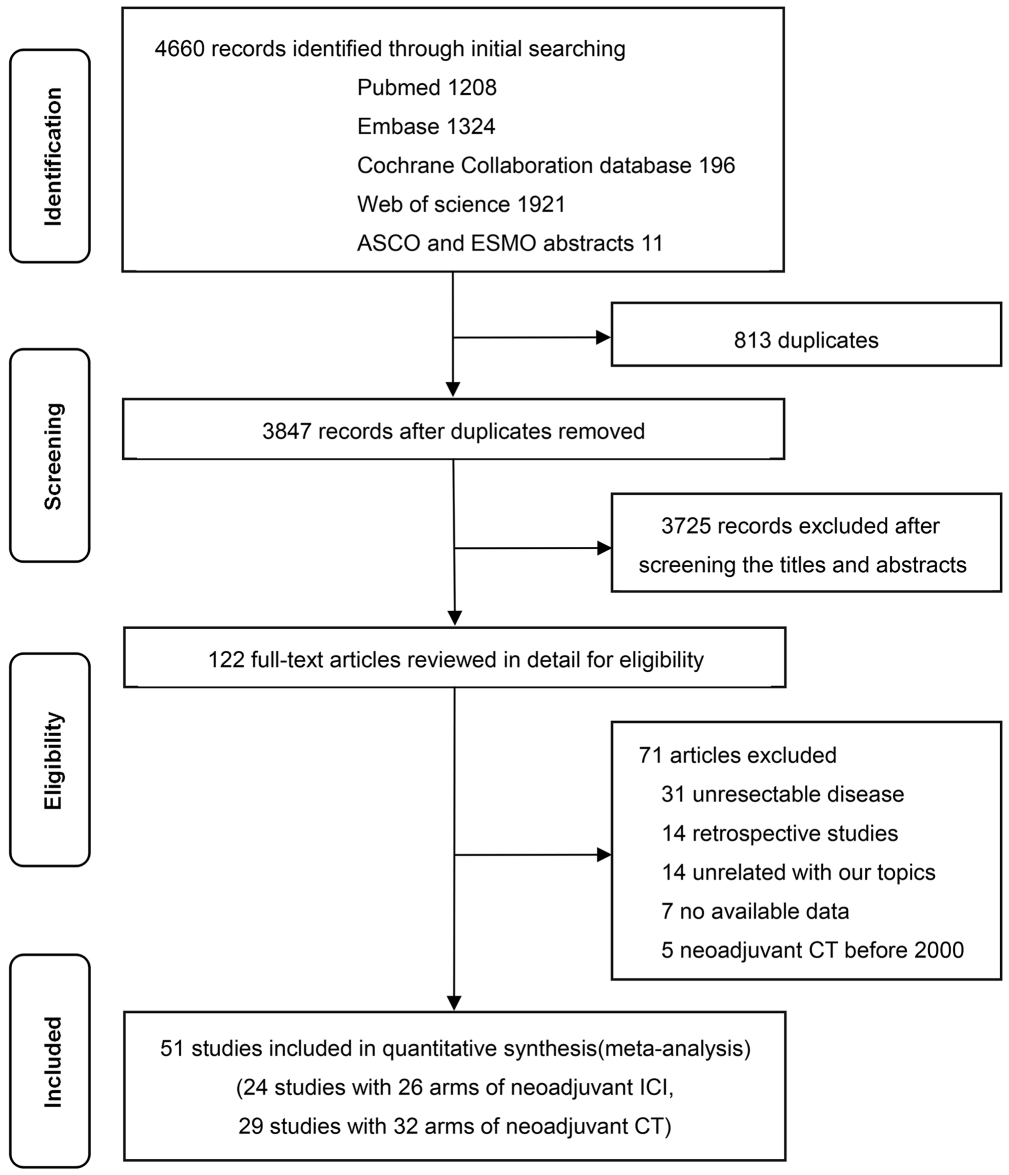
Literature search and selection. ICI, checkpoint inhibitor; CT, chemotherapy.

Following the search strategy, 4,660 studies were identified in the initial search. After screening the abstract and/or full text, 4,609 studies were excluded. Finally, 51 articles were eligible for inclusion. The selection process and reasons for study exclusion are shown in **Figure 1**. Among the 51 included trials, 24 studies (26 arms) with 1,043 patients examined nICI (5–28), and 29 studies (32 arms) with 2,337 patients examined nCT (13, 14, 35–61). The median patient ages were 65 years (interquartile range [IQR], 62–66 years) and 61 years (IQR, 58–64 years) for patients receiving nICI and nCT, respectively, and the median sample sizes were 30 participants (IQR, 17–39) and 47 participants (IQR, 31-88), respectively. The main characteristics and outcomes of included studies are presented in **Tables 1**, **2** and **Supplementary File: Table S3** for nICI and **Supplementary File: Tables S4** and **S5** for nCT.

**Table 1:** Characteristics of studies examining neoadjuvant ICI.

First author/year	Country	Phase (design)	size	Male (%)	Median age	SCC (%)	Stage 1/2/3(%)	ICI intervention*No. of cycles	Type of resection (%)^#^	Surgery time^&^
Bar/2019 (5)	Israel	I (single-arm)	10	60	NR	60	NR	Pembrolizumab*2	NR	2w
Besse/2020 (6)	France	II (single-arm)	30	50	64	17	50/20/30	Atezolizumab*1	NR	NR
Eichhorn/2021 (7)	Germany	II (single-arm)	15	47	59.8	13	0/40/60	Pembrolizumab*2	100/0/0	NR
Forde/2018 (8)	USA	II (single-arm)	22	48	67	29	19/48/33	Nivolumab*2	NR	2w
Gao/2021 (9)	China	Ib (single-arm)	40	82.5	62	82.5	20/35/45	Sintilimab*2	35/35/30	7-21d
Lee/2021 (10)	USA	II (single-arm)	181	49	65.1	38	9/41/50	Atezolizumab*1-2	79/9/12	10-73d
Tong/2021 (11)	USA	II (single-arm)	30	53	72	57	30/43/27	Pembrolizumab*2	72/12/16	7-35d
Wislez/2020 (12)	France	Single-arm	46	67.4	61	41	11/87/2	Durvalumab*3	67/20/13	2-14d
Forde/2021 (13)	USA	III (dual-arm)	179	72	64	49	23/14/63	Nivolumab+CT*3	76/17/19	≤ 6w
Lei/2020 (14)	China	II (dual-arm)	14	NR	NR	NR	0/0/100	Camrelizumab+CT*3	NR	NR
Provencio/2020 (15)	Spain	II (single-arm)	46	74	63	35	0/0/100	Nivolumab+CT*3	85/7/7	6-7w
Rothschild/2021 (16)	Switzerland	II (single-arm)	67	52	61	33	0/0/100	Durvalumab*2+CT*3	78/9/13	2-4w
Shen/2021 (17)	China	Single-arm	37	94.6	62.8	100	0/8/92	Pembrolizumab+CT*2	60/5/35	3-4w
Shu/2020 (18)	USA	II (single-arm)	30	50	67	40	0/23/77	Atezolizumab+CT*2-4	73/12/15	4w
Tfayli/2020 (19)	Lebanon	II (single-arm)	15	46.7	65	13.3	13/33/54	Avelumab*4+CT*3	NR	NR
Wang/2021 (20)	China	Single-arm	72	91.7	62.2	91.7	0/0/100	Anti-PD-1+CT*2	NR	3-5w
Yang/2017 (21)	USA	II (single-arm)	24	50	65	37	0/21/79	Ipilimumab*2+CT*3	76/8/16	≤ 12w
Zhao/2021 (22)	China	II (single-arm)	33	81.8	61	54.5	0/0/100	Toripalimab+CT*3	73/20/7	4–5w
Zinner/2020 (23)	USA	Single-arm	13	62	69	69	NR	Nivolumab+CT*3	NR	NR
Reuss/2020 (24)	USA	II (single-arm)	9	78	NR	11	11/22/67	Nivolumab*3 +Ipilimumab*1	NR	2w
Cascone/2021 (25)	USA	II (dual-arm)	23	65	66.1	43	48/30/22	Nivolumab*3	NR	3-6w
			21	62	65	33	57/24/19	Nivolumab*3 +Ipilimumab*1	NR	3-6w
Altorki/2021 (26)	USA	II (dual-arm)	30	53	71·0	37	37/16/47	Durvalumab*2	81/15/4	2–6w
			30	50	70·0	40	27/33/40	Durvalumab*2+SBRT	65/19/16	2–6w
Hong/2021 (27)	Korea	Ib (single-arm)	24	83	66	50	0/0/100	Durvalumab*2+CRT	NR	NR
Lemmon/2021 (28)	USA	I (single-arm)	9	33	66	NR	0/0/100	Pembrolizumab*3+CRT	NR	NR

ICI, checkpoint inhibitor; CT, chemotherapy; CRT, chemoradiotherapy; SBRT, stereotactic body radiotherapy; SCC,.squamous cell carcinoma; w, week; d, day; NR, not reported.

#Type of resection (lobectomy/pneumonectomy/others) (%).

&Surgery time (after the last dose of neoadjuvant therapy).

**Table 2:** Outcomes of studies of neoadjuvant ICI.

First author/year	ICI intervention	ORR	MPR rate	pCR rate	Surgical resection rate	R0 resection rate	Surgical complication	Grade 3–5 TRAEs
Bar/2019 (5)	Pembrolizumab	NR	40%	NR	100%	NR	NR	NR
Besse/2020 (6)	Atezolizumab	0%	0%	0%	100%	97%	10%	0%
Eichhorn/2021 (7)	Pembrolizumab	27%	27%	13%	100%	100%	7%	20%
Forde/2018 (8)	Nivolumab	10%	45%	15%	100%	100%	NR	5%
Gao/2021 (9)	Sintilimab	20%	41%	16%	93%	97%	11%	10%
Lee/2021 (10)	Atezolizumab	NR	19%	6%	88%	91%	3%	5%
Tong/2021 (11)	Pembrolizumab	NR	28%	12%	83%	88%	48%	3%
Wislez/2020 (12)	Durvalumab	9%	NR	NR	100%	89%	NR	0%
Forde/2021 (13)	Nivolumab+CT	54%	47%	30%	83%	83%	28%	19%
Lei/2020 (14)	Camrelizumab+CT	86%	86%	57%	100%	NR	NR	NR
Provencio/2020 (15)	Nivolumab+CT	76%	83%	63%	89%	100%	29%	30%
Rothschild/2021 (16)	Durvalumab+CT	58%	62%	18%	82%	93%	NR	13%
Shen/2021 (17)	Pembrolizumab+CT	86%	65%	46%	100%	100%	NR	11%
Shu/2020 (18)	Atezolizumab+CT	63%	59%	34%	97%	90%	NR	NR
Tfayli/2020 (19)	Avelumab+CT	27%	27%	9%	73%	NR	NR	27%
Wang/2021 (20)	Anti-PD-1+CT	94%	NR	29%	100%	NR	NR	NR
Yang/2017 (21)	Ipilimumab+CT	58%	NR	15%	54%	100%	NR	46%
Zhao/2021 (22)	Toripalimab+CT	88%	67%	50%	91%	97%	17%	9%
Zinner/2020 (23)	Nivolumab+CT	46%	85%	38%	100%	NR	NR	15%
Reuss/2020 (24)	Nivolumab+ipilimumab	11%	33%	33%	67%	NR	NR	33%
Cascone/2021 (25)	Nivolumab	22%	24%	10%	91%	100%	NR	13%
	Nivolumab+ipilimumab	19%	50%	38%	76%	100%	NR	10%
Altorki/2021 (26)	Durvalumab	3%	8%	0%	87%	88%	NR	20%
	Durvalumab+SBRT	47%	62%	31%	87%	96%	NR	23%
Hong/2021 (27)	Durvalumab+CRT	40%	78%	39%	75%	100%	NR	17%
Lemmon/2021 (28)	Pembrolizumab+CRT	75%	NR	67%	75%	100%	NR	NR

ICI, checkpoint inhibitor; CT, chemotherapy; CRT, chemoradiotherapy; SBRT, stereotactic body radiotherapy; ORR, objective response rate; MPR, major pathologic response; pCR, pathological complete response; TRAEs, treatment-related adverse events; NR, not reported.

### Assessment of included studies and publication bias

There were only two RCTs examining nICI vs. nCT which were rated with a low (13) or unclear risk of bias (14) (**Supplementary File: Figure S1**). As single-arm trials have a high risk of bias by their nature, they were not further assessed for bias. The RCTs examining nICI or nCT vs. other treatments were considered as single-arm studies because only the experimental arms were used in this study.

The funnel plots are presented in **Supplementary File: Figure S2**. The Begg’s and Egger’s test results indicated potential publication bias in MPR rate for nICI monotherapy (Egger test, P = 0.01), pCR rate for nCT (Egger test, P = 0.003; Begg’s test, P = 0.006), and grade ≥3 TRAEs for nICI combination (Begg’s test, P = 0.04) and nICI monotherapy (Egger test, P = 0.02; Begg’s test, P = 0.04).

### Outcomes of nICI vs. nCT

The results are summarized in **Figure 2**. The detailed forest plots are presented in **Supplementary File: Figures S3–S9**.

**Figure 2:**
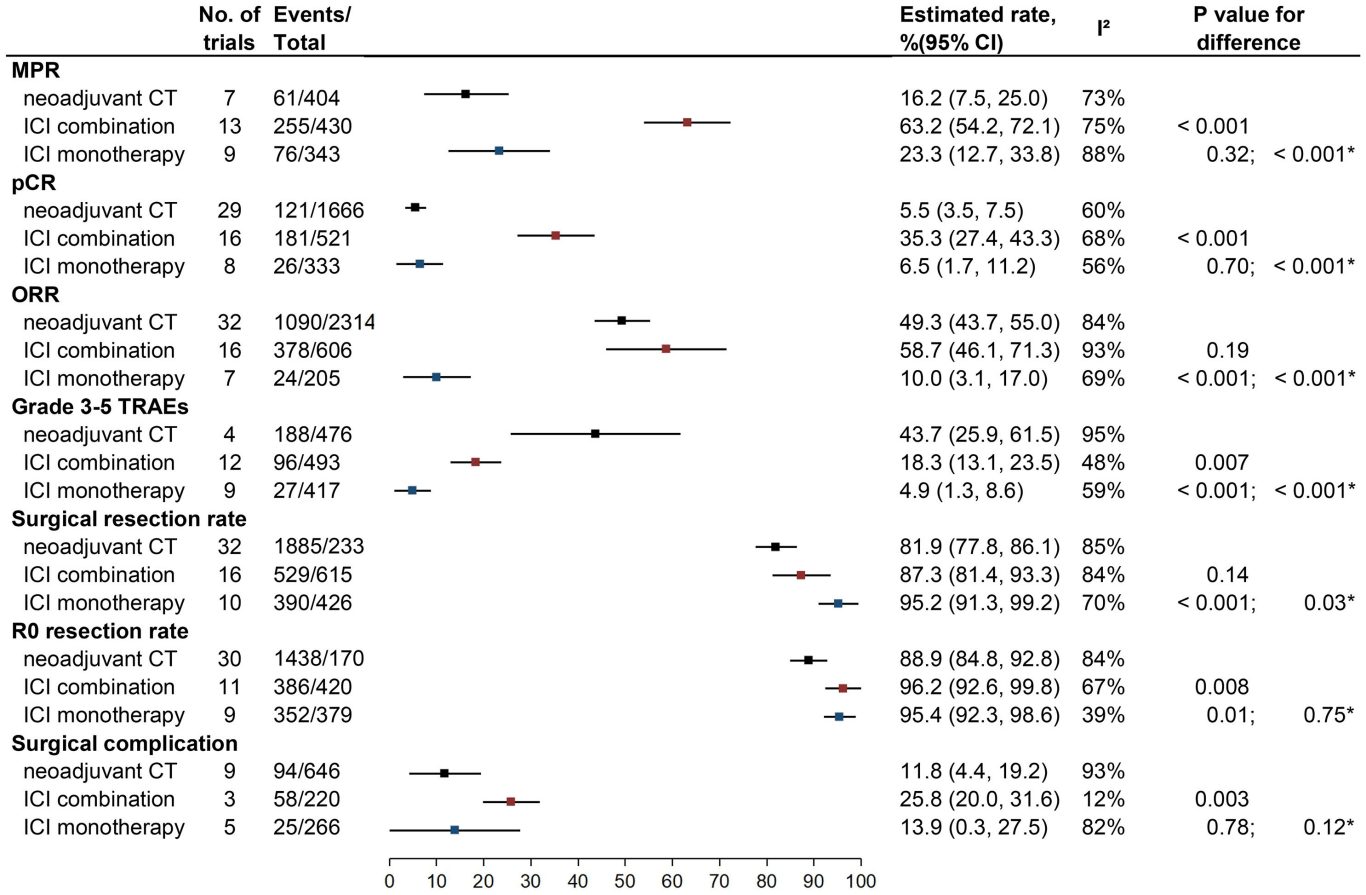
Outcomes of neoadjuvant CT vs. neoadjuvant ICI combination therapy vs. neoadjuvant ICI monotherapy. MPR, major pathologic response; pCR, pathological complete response; ORR, objective response rate; TRAEs, treatment-related adverse events; ICI, checkpoint inhibitor; CT, chemotherapy.

#### MPR

Twenty-two studies of nICI (13 of combination and nine of monotherapy) and seven studies of nCT reported rates of MPR. The estimated MPR rate was 63.2% (95% CI: 54.2%–72.1%; I^2^ = 75%) for nICI combination, which was higher than for nICI monotherapy (23.3%, 95% CI: 12.7%–33.8%, I^2^ = 88%; P < 0.001) and nCT (16.2%, 95% CI: 7.5%–25.0%; I^2^ = 73%; P < 0.001); there was no significant difference between nICI monotherapy and nCT (P = 0.32).

#### pCR

Twenty-four studies of nICI (16 of combination and eight of monotherapy) and 29 studies of nCT provided rates of pCR. The estimated pCR rate for nICI combination (35.3%, 95% CI: 27.4%–43.3%; I^2^ = 68%) was higher than for nICI monotherapy (6.5%, 95% CI: 1.7%–11.2%, I^2^ = 56%; P < 0.001) and nCT (5.5%, 95% CI: 3.5%–7.5%; I^2^ = 60%; P < 0.001); no significant difference was observed between nICI monotherapy and nCT (P = 0.70).

#### ORR

Twenty-three studies of nICI (16 of combination and seven of monotherapy) and 32 studies of nCT provided ORR. Either nICI combination (58.7%, 95% CI: 46.1%–71.3%; I^2^ = 93%) or nCT (49.3%, 95% CI: 43.7%–55%; I^2^ = 84%) achieved higher ORR than nICI monotherapy (10.0%, 95% CI: 3.1%–17.0%; I^2^ = 69%; P < 0.001 for each comparison); no significant difference was observed between nICI combination and nCT (P = 0.19).

#### Grade ≥3 TRAEs

Twenty-one studies of nICI (12 of combination and nine of monotherapy) and four studies of nCT reported incidence of the overall grade ≥3 TRAEs. Both nICI combination (18.3%, 95% CI: 13.1%–23.5%; I^2^ = 48%) and nICI monotherapy (4.9%, 95% CI: 1.3%–8.6%; I^2^ = 59%) had a lower incidence of grade ≥3 TRAEs than nCT (43.7%, 95% CI: 25.9%–61.6%; I^2^ = 95%; P = 0.007 and P < 0.001); the difference between nICI monotherapy and nICI combination was also significant (P < 0.001).

Thirteen studies of nICI combination (total 406 patients) and eight studies of nICI monotherapy (total 236 patients) reported individual grade ≥3 TRAEs (**Supplementary File: Table S6**). Of the 120 cases of grade ≥3 TRAEs that occurred in the nICI combination cohort, the leading cause was neutropenia (n = 30; 25.0%), followed by AST/ALT increase (n = 12; 10.0%), pneumonia/pneumonitis (n = 8; 6.7%), and fatigue (n = 8; 6.7%); three grade 5 TRAEs were observed (two cardiopulmonary events and one ARDS). Of the 21 cases of grade ≥3 TRAEs in the nICI monotherapy cohort, the leading cause was pneumonia/pneumonitis (n = 5; 23.8%), followed by hypokalemia (n = 3; 14.3%) and skin rash (n = 2; 9.5%); two grade 5 TRAEs were observed (one pneumonitis and one stroke).

#### Surgical resection rate

Twenty-six studies of nICI (16 of combination and 10 of monotherapy) and 32 studies of nCT reported surgical resection rate. nICI monotherapy achieved a higher surgical resection rate (95.2%, 95% CI: 91.3%–99.2%; I^2^ = 70%) compared with nICI combination (87.3%, 95% CI: 81.4%–93.3%, I^2^ = 84%; P = 0.03) and nCT (81.9%, 95% CI: 77.8%–86.1%, I^2^ = 85%; P < 0.001); there was no difference between nICI combination and nCT (P = 0.14).

#### R0 resection rate

Twenty studies of nICI (11 of combination and nine of monotherapy) and 30 studies of nCT reported the R0 resection rate. Both nICI combination (96.2%, 95% CI: 92.6%–99.8%, I^2^ = 67%) and nICI monotherapy (95.4%, 95% CI: 92.3%–98.6%, I^2^ = 39%) showed a higher R0 resection rate than nCT (88.9%, 95% CI: 84.8%–92.8%; I^2^ = 84%; P = 0.008 and P = 0.01); no difference was observed between nICI combination and nICI monotherapy (P = 0.75).

#### Surgical complication

Eight studies of nICI (three of combination and five of monotherapy) and nine studies of nCT reported incidence of surgical complication. Most of the studies did not detail whether surgical complication occurred within 30 or 90 days of surgery. Incidence of surgical complication for nICI combination (25.8%, 95% CI: 20.0%–31.6%; I^2^ = 12%) was similar with nICI monotherapy (13.9%, 95% CI: 0.3%–27.5%; I^2^ = 82%; P = 0.12) but was higher than nCT (11.8%, 95% CI: 4.4%–19.2%; I^2^ = 93%; P = 0.003); no difference was observed between nICI monotherapy and nCT (P = 0.78).

### Sensitivity analysis

When individual studies of nICI or nCT were removed one at a time from the analyses for MPR, pCR, and grade ≥3 TRAEs, the results were not markedly altered by any single study, indicating a good stability of these results (**Supplementary File: Figure S10**).

### nICI vs. nCT in studies published within the last 5 years

The results of nICI remained the same because all the studies were published within the last 5 years. As for nCT, there were only six studies included. MPR (16.2%) and pCR (2.4%) rates and ORR (49.3%) were similar with the results from all studies, while incidence of grade 3–5 TRAEs (20.7%) and surgical resection rate (67.8%) decreased obviously, and incidence of surgical complication (27.6%) increased obviously. However, only one and two studies reported the incidence of grade 3–5 TRAEs and surgical complication, respectively. The details are shown in **Supplementary File: Figure S11**.

### Subgroup analyses of MPR and pCR in patients receiving nICI

**Figure 3:**
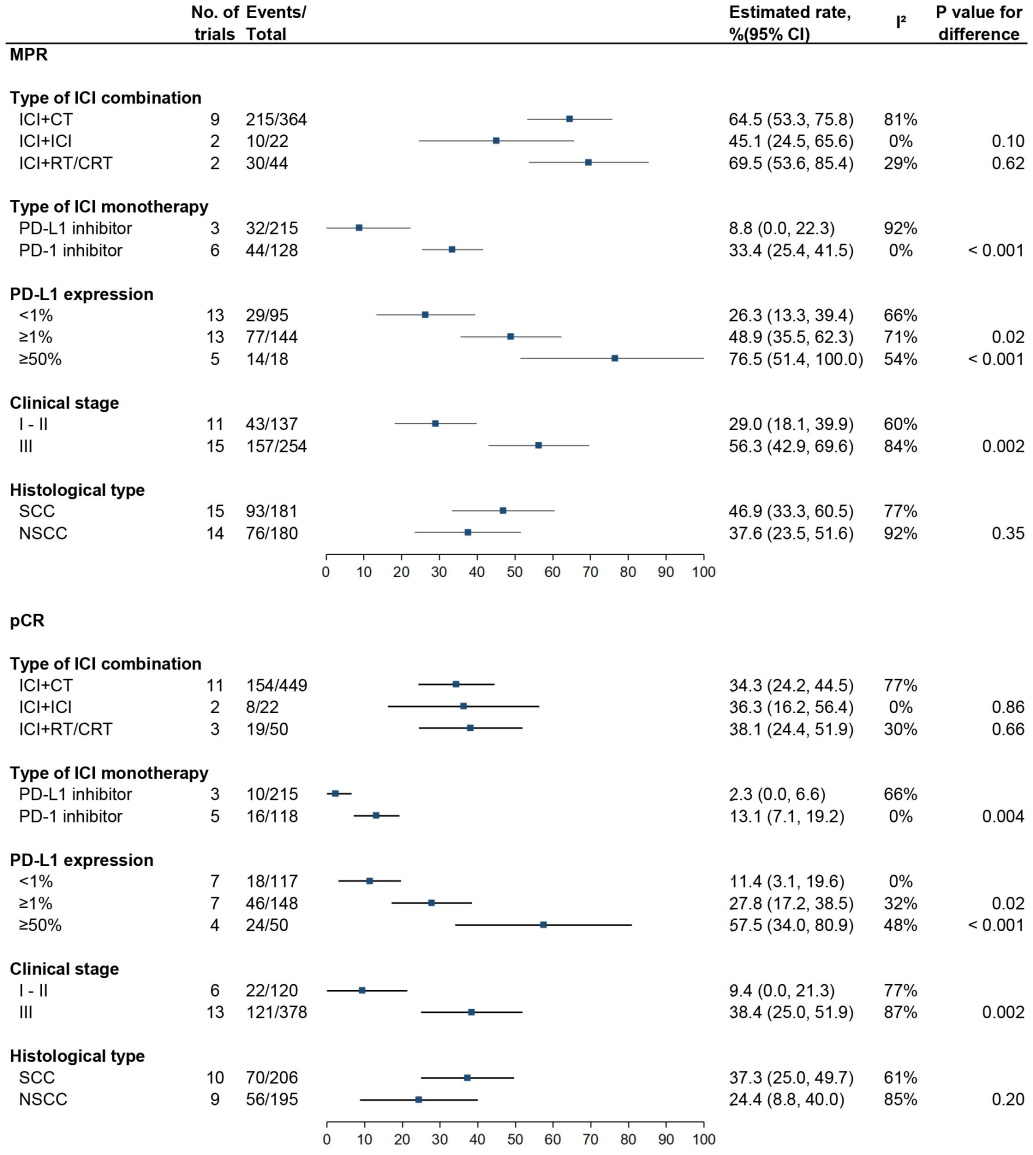
Subgroup analyses for MPR and pCR in patients receiving neoadjuvant ICI. MPR, major pathologic response; pCR, pathological complete response; ICI, checkpoint inhibitor; CT, chemotherapy; RT; radiotherapy; CRT; chemoradiotherapy; SCC, squamous cell carcinoma; NSCC, non-squamous cell carcinoma.

Results of the subgroup analyses are shown in **Figure 3**.

#### Type of nICI combination

There were no significant differences in either MPR or pCR rate between ICI plus chemotherapy (64.5%, 95% CI: 53.3%–75.8%, I^2^ = 81% and 34.3%, 95% CI: 24.2%–44.5%, I^2^ = 77%), dual ICI combination (45.1%, 95% CI: 24.5%–65.6%, I^2^ = 0% and 36.3%, 95% CI: 16.2%–56.4%, I^2^ = 0%), and ICI plus radiotherapy or chemoradiotherapy (RT/CRT) (69.5%, 95% CI: 53.6%–85.4%, I^2^ = 29% and 38.1%, 95% CI: 24.4%–51.9%, I^2^ = 30%) (P > 0.05 for each comparison).

#### Type of nICI monotherapy

Compared with PD-L1 inhibitor, PD-1 inhibitor was associated with higher MPR (33.4%, 95% CI: 25.4%–41.5%, I^2^ = 0% vs. 8.8%, 95% CI: 0.0%–22.3%, I^2^ = 92%; P < 0.001) and pCR (13.1%, 95% CI: 7.1%–19.2%, I^2^ = 0% vs. 2.3%, 95% CI: 0.0%–6.6%, I^2^ = 66%; P = 0.004) rates.

#### PD-L1 expression

MPR rates were 26.3% (95% CI: 13.3%–39.4%, I^2^ = 66%), 48.9% (95% CI: 35.5%–62.3%, I^2^ = 71%; P = 0.02), and 76.5% (95% CI: 51.4%–100.0%, I^2^ = 54%; P < 0.001) for patients with PD-L1 expression <1%, ≥1%, and ≥50%, respectively, and pCR rates were 11.4% (95% CI: 3.1%–19.6%, I^2^ = 0%), 27.8% (95% CI: 17.2%–38.5%, I^2^ = 32%; P = 0.02), and 57.5% (95% CI: 34.0%–80.9%, I^2^ = 48%; P = < 0.001), respectively.

#### Clinical stage

MPR rates were 29.0% (95% CI: 18.1%–39.9%, I^2^ = 60%) for stages 1–2 vs. 56.3% (95% CI: 42.9%–69.6%, I^2^ = 84%; P = 0.002) for stage 3, and pCR rates were 9.4% (95% CI: 0.0%–21.3%, I^2^ = 77%) vs. 38.4% (95% CI: 25.0%–51.9%, I^2^ = 87%; P = 0.002).

Further analysis according to treatment mode showed that differences in MPR and pCR rates between stages 1–2 and stage 3 were observed only in patients receiving nICI combination (P = 0.002 and P = 0.03) but not in patients receiving nICI monotherapy (P = 0.37 and P = 0.34) (**Supplementary File: Figure S12**).

#### Histological type

There were no significant differences in MPR (46.9%, 95% CI: 33.3%–60.5%, I^2^ = 77% vs. 37.6%, 95% CI: 23.5%–51.6%, I^2^ = 92%; P = 0.35) and pCR (37.3%, 95% CI: 25.0%–49.7%, I^2^ = 61% vs. 24.4%, 95% CI: 8.8%–40.0%, I^2^ = 85%; P = 0.20) rates between patients with squamous cell carcinoma and non-squamous cell carcinoma.

## Discussion

This is a comprehensive systematic review and meta-analysis assessing MPR and pCR rates and safety of nICI (monotherapy and combination) and nCT in patients with resectable NSCLC. It showed that nICI combination therapy was associated with higher MPR (63.2% vs. 16.2%, P < 0.001) and pCR (35.3% vs. 5.5%, P < 0.001) rates compared to nCT. As for safety, nICI combination had a similar surgical resection rate and higher R0 resection rate than nCT. In addition, we also found a lower incidence of grade 3–5 TRAEs and more surgical complication of nICI combination vs. nCT. However, there were only four studies of nCT providing data of grade 3–5 TRAEs and three studies of nICI combination providing data of surgical complication, making the results less reliable. Thus, the two results should be interpreted with caution. Moreover, it should be noted that the time periods of the nCT trials included are over 20 years dating from the 2000s, while all trials of nICI are within the last 5 years. During these 20 years, the management of resectable NSCLC has changed a lot in terms of pre-therapeutic workup, surgical technique chemotherapy regimen, etc. To make the comparison more reasonable, we performed a subgroup analysis in studies published within the last 5 years. However, we found similar results of MPR and pCR rates with those from all studies, further supporting the better antitumor activity of nICI combination vs. nCT.

Regarding nICI, nICI combination therapy had higher MPR and pCR rates than nICI monotherapy (23.3%, P < 0.001 and 6.5%, P < 0.001), but with more grade ≥3 TRAEs and a lower surgical resection rate. The less tolerability of nICI combination may limit its use in some special patients (such as elderly patients or those with poor performance). In this case, nICI monotherapy may play a role due to the best tolerability among the three neoadjuvant treatments and the comparable MPR and pCR rates with nCT, while which class of ICI (PD-1 or PD-L1 inhibitor) is better remains unclear. In our meta-analysis, PD-1 inhibitor achieved higher MPR (33.4% vs. 8.8%, P < 0.001) and pCR (13.1% vs. 2.3%, P = 0.004) rates than PD-L1 inhibitor, suggesting a better selection of PD-1 inhibitor when using ICI monotherapy as a neoadjuvant treatment.

ICI plus chemotherapy was the most common regimen tested in clinical trials and had been reported to be associated with high MPR and/or pCR rates and acceptable toxicity (13–18, 22, 23). In addition, dual-ICI combination (nivolumab plus ipilimumab) and ICI plus RT/CRT were also examined in several recent phase 1 or 2 studies (24–28). Although the two regimens also showed promising MPR and/or pCR rates, their safety was still a concern due to the opposite results reported in individual studies. As for the dual-ICI combination, grade 3–5 TRAEs were only 10% (2/21) in the NEOSTAR study (25) but was 33% (3/9) in the trial by Reuss et al. (24) which led to a decision to terminate the study early. With regard to ICI plus RT/CRT, a phase 2 trial of durvalumab plus CRT (27) reported acceptable grade 3–5 TRAEs of 16.7%, while a phase 1 trial of pembrolizumab plus CRT (28) showed that the serious adverse events were 100% (9/9) including two grade 5 events which met the stopping rule for safety. In our meta-analysis, MPR and pCR rates were similar between the three ICI combination regimens. Nevertheless, the value of dual-ICI combination and ICI plus RT/CRT in the neoadjuvant setting needs further evaluation due to inconsistent safety results in individual studies. Currently, ICI plus chemotherapy is likely to be the optimal nICI combination strategy.

Since pathologic response can be assessed only after surgical resection, exploring biomarkers in the selection of patients who may benefit from nICI upfront is important. PD-L1 expression has been demonstrated to be an important predictive biomarker for ICI efficacy in metastatic NSCLC, while its predictive role for tumor pathologic response in the neoadjuvant setting is under evaluation. In the present meta-analysis, MPR and PCR rates for patients with PD-L1 ≥1% (48.9% and 27.8%) and ≥50% (76.5% and 57.5%) were significantly higher than for patients with PD-L1 <1% (26.3% and 11.4%), suggesting a positive correlation between tumor pathologic response rate and PD-L1 level. Other biomarkers such as tumor mutational burden (TMB) (8), ctDNA (13), tumor-infiltrating lymphocytes (TILs) (25), and immune-related genes (62) were also reported to be correlated with MPR. Due to limited data, their predictive role needs to be further explored.

Besides predictive biomarkers, patient characteristics such as clinical stage and histological type have also been reported to be possible predictors of nICI. In terms of clinical stage, nivolumab plus chemotherapy achieved a promising MPR rate of 85% and a pCR rate of 58.5% for stage 3A patients in the NADIM trial (15). A low proportion of residual viable tumor cells was observed for patients with stage 3 compared to patients with stages 1–2 (8% vs. 28%) in the nivolumab plus chemotherapy group in the CheckMate 816 study (13). In our meta-analysis, superior MPR and pCR rates for stage 3 vs. 1–2 were observed in patients receiving nICI combination therapy but not in those receiving nICI monotherapy, supporting the possibility that nICI combination therapy had more antitumor efficacy for patients with stage 3. Nevertheless, the findings need to be validated in large RCTs, and the mechanism also needs to be explored. As for histological type, squamous cell NSCLC exhibited a superior MPR compared with adenocarcinoma in two trials (9, 18), possibly due to greater baseline tumor necrosis in squamous cell carcinomas (18). However, there were opposite results from the CheckMate 159 study (8) showing that adenocarcinoma had a higher MPR of 46.2% compared with squamous cell NSCLC at 33.3%. In our study, no significant differences in MPR and pCR rates were observed according to histological type. Thus, it is still hard to draw a conclusion that squamous cell NSCLC would benefit more from nICI.

CT imaging is traditionally used to assess the tumor response after treatment. However, a recent study of nCT showed that there was no relationship between CT RECIST response and pathologic response in NSCLC patients (63). This phenomenon was also observed for patients receiving nICI. In our meta-analysis, we found that most of individual studies of nICI reported a higher MPR rate than ORR (**Supplementary File: Figure S13**), suggesting the poor predictive role of CT imaging for the pathologic response. In a recent phase 2 study (64) using emission tomography-CT (PET-CT) to evaluate tumor response to nICI, maximum standardized uptake value (SUVmax) reduction after sintilimab was significantly correlated with pathologic response. Nevertheless, the predictive value of PET-CT needs to be investigated in more studies.

Several previous meta-analyses (65–70) have also evaluated neoadjuvant immunotherapy in NSCLC (**Supplementary File: Table S7**). Among them, the largest two studies (65, 66) which were published in 2022 included 21 trials with 792 patients (65) and 15 trials with 809 patients (66), respectively. The two studies assessed outcomes of MPR, pCR, ORR, TRAEs, and surgical safety of nICI and conducted subgroup analyses according to area, arms, nICI modes, and ICI types (65) or according to nICI modes, ICI types, PD-L1 expression, histology, and smoking (66). Compared to the previous meta-analyses, our study included more trials and more sample sizes (24 trials with 1,043 patients). In addition, several additional subgroup analyses such as PD-L1 ≥50%, clinical stages of 1–2 and 3, and nICI modes of ICI plus RT/CRT were conducted in our study, and with new findings. Moreover, we also collected data from eligible studies examining nCT (29 trials with 2,337 patients) and made a comparison with that from nICI. Thus, our meta-analysis would be more comprehensive in evaluating the value of nICI in resectable NSCLC.

There are several limitations in the current study. First, due to that most of included trials of nICI had a short follow-up time without mature OS data, we used MPR and pCR as the primary outcomes of interest. Although there is evidence supporting MPR and/or pCR being predictive for OS in resectable NSCLC, most of the data are from nCT (29, 30), and their predictive value might vary according to type of neoadjuvant therapy. For example, in several studies examining neoadjuvant chemoradiotherapy vs. chemotherapy (47, 57), the increased MPR rate of chemoradiotherapy did not translate to improved OS. One possible explanation is that the MPR in this setting represented the local cytotoxic effect of radiotherapy but did not reflect control of micrometastases by chemotherapy (71). Thus, there is still uncertainty to the use of MPR or pCR as a surrogate endpoint of OS in patients receiving nICI due to lack of studies assessing the correlation. Second, this is a single-arm-based meta-analysis, and the findings are hypothesis-generating. Lack of large head-to-head RCTs prevents us from making a firm conclusion. Finally, there are publication bias and substantial heterogeneity among studies. By subgroup analyses, we found that type of nICI combination, nICI class, and PD-L1 expression might account for some heterogeneity for MPR and/or pCR.

## Conclusions

nCI combination therapy is associated with higher MPR and pCR rates compared to nCT and nICI monotherapy, and with acceptable tolerability. PD-L1 status appears to be predictive of MPR and pCR in patients receiving nICI. PD-1 inhibitor appears to be superior to PD-L1 inhibitor. Patients with stage 3 seem to benefit more from nICI combination therapy than patients with stages 1–2. Nevertheless, these findings are hypothesis-generating and require further validation by large RCTs. Moreover, future trials of nICI with long-term survival outcomes are wanted to clarify the correlation between MPR and overall survival.

## Data availability statement

The original contributions presented in the study are included in the article/**Supplementary Material**. Further inquiries can be directed to the corresponding author.

## Ethics statement

Ethical review and approval was not required for the study on human participants in accordance with the local legislation and institutional requirements. The ethics committee waived the requirement of written informed consent for participation.

## Author contributions

Conception and design: JD. Collection and assembly of data: HW and TL. Data analysis and interpretation: all authors. Manuscript writing: all authors. Final approval of manuscript: all authors.

## Conflict of interest

The authors declare that the research was conducted in the absence of any commercial or financial relationships that could be construed as a potential conflict of interest.

## Publisher’s note

All claims expressed in this article are solely those of the authors and do not necessarily represent those of their affiliated organizations, or those of the publisher, the editors and the reviewers. Any product that may be evaluated in this article, or claim that may be made by its manufacturer, is not guaranteed or endorsed by the publisher.
