# Supplementary material for: Neoadjuvant immunotherapy and neoadjuvant chemotherapy in resectable non-small cell lung cancer: A systematic review and single-arm meta-analysis
Source: Front Oncol. 2022 Sep 21;12:901494. doi: 10.3389/fonc.2022.901494 (PMC9533019; doi:10.3389/fonc.2022.901494)
Supplement: Supplementary file 1 [file DataSheet_1.doc]

**Table S1 PRISMA Checklist**

| **Section/Topic** | **Item #** | **Checklist Item** | **Reported on Page #** |
| --- | --- | --- | --- |
| **TITLE** |  |  |  |
| Title | 1 | Identify the report as a systematic review incorporating a meta-analysis (or related form of meta-analysis). | 1 |
| **ABSTRACT** |  |  |  |
| Structured summary | 2 | Provide a structured summary including, as applicable:  **Background:** main objectives  **Methods:** data sources; study eligibility criteria, participants, and interventions; study appraisal; and synthesis methods, such as network meta-analysis.  **Results:** number of studies and participants identified; summary estimates with corresponding confidence/credible intervals; treatment rankings may also be discussed. Authors may choose to summarize pairwise comparisons against a chosen treatment included in their analyses for brevity.  **Discussion/Conclusions:** limitations; conclusions and implications of findings.  **Other:** primary source of funding; systematic review registration number with registry name. | 2-3 |
| **INTRODUCTION** |  |  |  |
| Rationale | 3 | Describe the rationale for the review in the context of what is already known, including mention of why a network meta-analysis has been conducted. | 4 |
| Objectives | 4 | Provide an explicit statement of questions being addressed, with reference to participants, interventions, comparisons, outcomes, and study design (PICOS). | 4-5 |
| **METHODS** |  |  |  |
| Protocol and registration | 5 | Indicate whether a review protocol exists and if and where it can be accessed (e.g., Web address); and, if available, provide registration information, including registration number. | CRD42021278661 |
| Eligibility criteria | 6 | Specify study characteristics (e.g., PICOS, length of follow-up) and report characteristics (e.g., years considered, language, publication status) used as criteria for eligibility, giving rationale. Clearly describe eligible treatments included in the treatment network, and note whether any have been clustered or merged into the same node (with justification). | 5-6 |
| Information sources | 7 | Describe all information sources (e.g., databases with dates of coverage, contact with study authors to identify additional studies) in the search and date last searched. | 5 |
| Search | 8 | Present full electronic search strategy for at least one database, including any limits used, such that it could be repeated. | Supplementary Table S2 |
| Study selection | 9 | State the process for selecting studies (i.e., screening, eligibility, included in systematic review, and, if applicable, included in the meta-analysis). | 5 |
| Data collection process | 10 | Describe method of data extraction from reports (e.g., piloted forms, independently, in duplicate) and any processes for obtaining and confirming data from investigators. | 6 |
| Data items | 11 | List and define all variables for which data were sought (e.g., PICOS, funding sources) and any assumptions and simplifications made. | 6 |
| **Geometry of the network** | **S1** | Describe methods used to explore the geometry of the treatment network under study and potential biases related to it. This should include how the evidence base has been graphically summarized for presentation, and what characteristics were compiled and used to describe the evidence base to readers. | NA |
| Risk of bias within individual studies | 12 | Describe methods used for assessing risk of bias of individual studies (including specification of whether this was done at the study or outcome level), and how this information is to be used in any data synthesis. | 6 |
| Summary measures | 13 | State the principal summary measures (e.g., risk ratio, difference in means). Also describe the use of additional summary measures assessed, such as treatment rankings and surface under the cumulative ranking curve (SUCRA) values, as well as modified approaches used to present summary findings from meta-analyses. | 7 |
| Planned methods of analysis | 14 | Describe the methods of handling data and combining results of studies for each network meta-analysis. This should include, but not be limited to:   - Handling of multi-arm trials; - Selection of variance structure; - Selection of prior distributions in Bayesian analyses; and - Assessment of model fit. | 7 |
| **Assessment of Inconsistency** | **S2** | Describe the statistical methods used to evaluate the agreement of direct and indirect evidence in the treatment network(s) studied. Describe efforts taken to address its presence when found. | NA |
| Risk of bias across studies | 15 | Specify any assessment of risk of bias that may affect the cumulative evidence (e.g., publication bias, selective reporting within studies). | 7-8 |
| Additional analyses | 16 | Describe methods of additional analyses if done, indicating which were pre-specified. This may include, but not be limited to, the following:   - Sensitivity or subgroup analyses; - Meta-regression analyses; - Alternative formulations of the treatment network; and - Use of alternative prior distributions for Bayesian analyses (if applicable). | 7 |
| **RESULTS** |  |  |  |
| Study selection | 17 | Give numbers of studies screened, assessed for eligibility, and included in the review, with reasons for exclusions at each stage, ideally with a flow diagram. | 8 |
| **Presentation of network structure** | **S3** | Provide a network graph of the included studies to enable visualization of the geometry of the treatment network. | NA |
| **Summary of network geometry** | **S4** | Provide a brief overview of characteristics of the treatment network. This may include commentary on the abundance of trials and randomized patients for the different interventions and pairwise comparisons in the network, gaps of evidence in the treatment network, and potential biases reflected by the network structure. | NA |
| Study characteristics | 18 | For each study, present characteristics for which data were extracted (e.g., study size, PICOS, follow-up period) and provide the citations. | Table 1 |
| Risk of bias within studies | 19 | Present data on risk of bias of each study and, if available, any outcome level assessment. | 8-9 |
| Results of individual studies | 20 | For all outcomes considered (benefits or harms), present, for each study: 1) simple summary data for each intervention group, and 2) effect estimates and confidence intervals. Modified approaches may be needed to deal with information from larger networks. | 9-14 |
| Synthesis of results | 21 | Present results of each meta-analysis done, including confidence/credible intervals. In larger networks, authors may focus on comparisons versus a particular comparator (e.g. placebo or standard care), with full findings presented in an appendix. League tables and forest plots may be considered to summarize pairwise comparisons. If additional summary measures were explored (such as treatment rankings), these should also be presented. | 9-14 |
| **Exploration for inconsistency** | **S5** | Describe results from investigations of inconsistency. This may include such information as measures of model fit to compare consistency and inconsistency models, P values from statistical tests, or summary of inconsistency estimates from different parts of the treatment network. | NA |
| Risk of bias across studies | 22 | Present results of any assessment of risk of bias across studies for the evidence base being studied. | 8-9 |
| Results of additional analyses | 23 | Give results of additional analyses, if done (e.g., sensitivity or subgroup analyses, meta-regression analyses, alternative network geometries studied, alternative choice of prior distributions for Bayesian analyses, and so forth). | 12-14 |
| **DISCUSSION** |  |  |  |
| Summary of evidence | 24 | Summarize the main findings, including the strength of evidence for each main outcome; consider their relevance to key groups (e.g., healthcare providers, users, and policy-makers). | 14-19 |
| Limitations | 25 | Discuss limitations at study and outcome level (e.g., risk of bias), and at review level (e.g., incomplete retrieval of identified research, reporting bias). Comment on the validity of the assumptions, such as transitivity and consistency. Comment on any concerns regarding network geometry (e.g., avoidance of certain comparisons). | 19 |
| Conclusions | 26 | Provide a general interpretation of the results in the context of other evidence, and implications for future research. | 19-20 |
| **FUNDING** |  |  |  |
| Funding | 27 | Describe sources of funding for the systematic review and other support (e.g., supply of data); role of funders for the systematic review. This should also include information regarding whether funding has been received from manufacturers of treatments in the network and/or whether some of the authors are content experts with professional conflicts of interest that could affect use of treatments in the network. | NA |

PICOS = population, intervention, comparators, outcomes, study design.

**Table S2** Search strategy

**a:** Search strategy in PubMed

| # | Query |
| --- | --- |
| #1 | "Lung Neoplasms”[mh] |
| #2 | Lung Neoplasms[tiab] OR Neoplasms, Lung[tiab] OR Lung Neoplasm[tiab] OR Neoplasm, Lung[tiab] OR Neoplasms, Pulmonary[tiab] OR Neoplasm, Pulmonary[tiab] OR Pulmonary Neoplasm[tiab] OR Pulmonary Neoplasms[tiab] OR Lung Cancer[tiab] OR Cancer, Lung[tiab] OR Cancers, Lung[tiab] OR Lung Cancers[tiab] OR Pulmonary Cancer[tiab] OR Cancer, Pulmonary[tiab] OR Cancers, Pulmonary[tiab] OR Pulmonary Cancers[tiab] OR Cancer of the Lung[tiab] OR Cancer of Lung[tiab] |
| #3 | "Carcinoma, Non-Small-Cell Lung"[mh] |
| #4 | Carcinoma, Non Small Cell Lung[tiab] OR Carcinomas, Non-Small-Cell Lung[tiab] OR Lung Carcinoma, Non-Small-Cell[tiab] OR Lung Carcinomas, Non-Small-Cell[tiab] OR Non-Small-Cell Lung Carcinomas[tiab] OR Non-Small-Cell Lung Carcinoma[tiab] OR Non Small Cell Lung Carcinoma[tiab] OR Carcinoma, Non-Small Cell Lung[tiab] OR Non-Small Cell Lung Cancer[tiab] OR NSCLC[tiab] |
| #5 | #1 OR #2 OR #3 OR #4 |
| #6 | "Neoadjuvant Chemotherapy"[mh] |
| #7 | Neoadjuvant Chemotherapy[tiab] OR Chemotherapy, Neoadjuvant[tiab] OR Neoadjuvant Chemotherapies[tiab] OR Chemotherapy Treatment, Neoadjuvant[tiab] OR Preoperative Chemotherapy[tiab] OR Pre-operative Chemotherapy[tiab] OR Induction Chemotherapy[tiab] |
| #8 | “Immunotherapy”[mh] |
| #9 | Immunotherapies[tiab] OR Programmed death ligand 1[tiab] OR PD-L1[tiab] OR Programmed death 1[tiab] OR PD-1[tiab] OR Anti-Programmed death ligand 1[tiab] OR Anti–PD-L1[tiab] OR Anti-Programmed death 1[tiab] OR Anti–PD-1[tiab] OR Atezolizumab[tiab] OR Durvalumab[tiab] OR Nivolumab[tiab] OR Pembrolizumab[tiab] OR Camrelizumab[tiab] OR Sintilimab[tiab] OR Cemiplimab[tiab] OR Tislelizumab[tiab] OR Ipilimumab[tiab] OR Avelumab[tiab] OR tremelimumab[tiab] |
| #10 | #6 OR #7 OR #8 OR #9 |
| #11 | Randomized Controlled Trial[pt] |
| #12 | Controlled Clinical Trial[pt] |
| #13 | Randomized[tiab] |
| #14 | Placebo[tiab] |
| #15 | Randomly[tiab] |
| #16  #17 | Trial[tiab]  Drug Therapy[sh] |
| #18 | Groups[tiab] |
| #19 | #11 OR #12 OR #13 OR #14 OR #15 OR #16 OR #17 OR #18 |
| #20 | Animals[mh] |
| #21 | Humans[mh] |
| #22 | #20 NOT #21 |
| #23 | #19 NOT #22 |
| #24 | #5 AND #10 AND #23 |

**b:** Search strategy in Embase

| # | Query |
| --- | --- |
| #1 | ‘lung cancer’/exp |
| #2 | ‘non small cell lung cancer’/exp |
| #3 | 'non small cell lung cancer':ab,ti OR ‘nsclc’:ab,ti |
| #4 | #1 OR #2 OR #3 |
| #5 | ‘immunotherapy'/exp |
| #6 | 'neoadjuvant chemotherapy'/exp |
| #7 | ‘programmed death ligand 1’:ab,ti OR ‘PD-L1’:ab,ti OR ‘programmed death 1’:ab,ti OR ‘PD-1’:ab,ti OR ‘anti-programmed death ligand 1’:ab,ti OR ‘anti–PD-L1’:ab,ti OR ‘anti-programmed death 1’:ab,ti OR ‘anti–PD-1’:ab,ti OR ‘atezolizumab’:ab,ti OR ‘durvalumab’:ab,ti OR ‘nivolumab’:ab,ti OR ‘pembrolizumab’:ab,ti OR ‘camrelizumab’:ab,ti OR ‘sintilimab’:ab,ti OR ‘cemiplimab’:ab,ti OR ‘avelumab’:ab,ti OR ‘tislelizumab’:ab,ti OR ‘anti–cytotoxic T-lymphocyte antigen 4’:ab,ti OR ‘anti–CTLA-4’:ab,ti OR ‘ipilimumab’:ab,ti OR ‘tremelimumab’:ab,ti OR ‘immunotherapy’:ab,ti OR ‘immune checkpoint inhibitors’:ab,ti OR ‘ICI’:ab,ti |
| #8 | 'preoperative chemotherapy':ab,ti OR ‘neoadjuvant chemotherapy’:ab,ti OR ‘chemotherapy, neoadjuvant’:ab,ti OR ‘neoadjuvant chemotherapies’:ab,ti OR ‘pre-operative chemotherapy’:ab,ti OR ‘induction chemotherapy’:ab,ti |
| #9 | #5 OR #6 OR #7 OR #8 |
| #10 | 'trial':ab,ti |
| #11 | 'random*':ab,ti |
| #12 | 'clinical trial'/de OR 'controlled clinical trial'/de OR 'randomized controlled trial'/de |
| #13 | #10 OR #11 OR #12 |
| #14 | #4 AND #9 AND #13 |

**c:** Search strategy in Cochrane Library

| # | Query |
| --- | --- |
| #1 | MeSH descriptor: [Carcinoma, Non-Small-Cell Lung] explode all trees |
| #2 | MeSH descriptor: [Lung Neoplasms] explode all trees |
| #3 | ((lung OR pulmon*) AND (neoplas* OR cancer OR carcinoma* OR tumour* or tumor*)) |
| #4 | (non-small cell*) OR (non small cell*) OR (nonsmall cell*) OR (NSCLC) |
| #5 | #1 OR #2 OR #3 OR #4 |
| #6 | MeSH descriptor: [Immunotherapy] explode all trees |
| #7 | (programmed death ligand 1 OR PD-L1 OR programmed death 1 OR PD-1 OR anti-programmed death ligand 1 OR anti–PD-L1 OR anti-programmed death 1 OR anti–PD-1 OR atezolizumab OR durvalumab OR nivolumab OR pembrolizumab OR camrelizumab OR sintilimab OR cemiplimab OR avelumab OR tislelizumab OR anti–cytotoxic T-lymphocyte antigen 4 OR anti–CTLA-4 OR ipilimumab OR tremelimumab OR immunotherapy OR immune checkpoint inhibitors OR ICI):ti,ab |
| #8 | (preoperative chemotherapy OR neoadjuvant chemotherapy OR chemotherapy, neoadjuvant OR neoadjuvant chemotherapies OR pre-operative chemotherapy OR induction chemotherapy):ti,ab |
| #9 | #6 OR #7 OR #8 |
| #10 | #5 AND #9 |

**d:** Search strategy in Web of Science

| # | Query |
| --- | --- |
| #1 | TS=("lung cancer" OR "non-small cell lung cancer" OR NSCLC OR ((lung OR pulmon*) AND (neoplas* OR cancer OR carcinoma* OR tumour* or tumor*))) |
| #2 | TS=("neoadjuvant Chemo*” OR “preoperative chemotherapy” OR “neoadjuvant chemotherapy” OR “chemotherapy, neoadjuvant” OR “neoadjuvant chemotherapies” OR “pre-operative chemotherapy” OR “induction chemotherapy”) |
| #3 | TS=(“programmed death ligand 1” OR “PD-L1” OR “programmed death 1” OR “PD-1” OR “anti-programmed death ligand 1” OR “anti–PD-L1” OR “anti-programmed death 1” OR “anti–PD-1” OR “atezolizumab” OR “durvalumab” OR “nivolumab” OR “pembrolizumab” OR “camrelizumab” OR “sintilimab” OR “tislelizumab” OR “cemiplimab” OR “avelumab”) |
| #4 | TS=(“anti–cytotoxic T-lymphocyte antigen 4” OR “anti–CTLA-4” OR “ipilimumab” OR “tremelimumab”) |
| #5 | TS=(“immunotherapy” OR “immune checkpoint inhibitors” OR “ICI”) |
| #6 | #2 OR #3 OR #4 OR #5 |
| #7 | TS=("randomized controlled trial" OR "controlled clinical trial" OR "clinical trial" OR "random*" OR "rct*" OR "crossover" OR "masked” OR “blind*" OR "placebo*") |
| #8 | #1 AND #6 AND #7 |

**Table S3 Survival outcomes of studies examining neoadjuvant ICI**

| First Author/Year | ICI intervention | Survival outcomes |
| --- | --- | --- |
| Forde/2018[8] | nivolumab | 18-m RFS: 73% |
| Gao/2021[9] | sintilimab | 2-y OS: 87.5%; 2-y OS: 91.7%(R0); median OS: 26.4m.  1-y DFS: 91.7%(R0); 2-y DFS: 73.3%(R0); median DFS: 23.9m. |
| Wislez/2020[12] | durvalumab | 18-m OS: 88.7%; 18-m RFS: 69.7%. |
| Provencio/2020[15] | nivolumab+CT | 12-m OS: 97.8%; 18-m OS: 93.5%; 2-y OS: 89.9%.  12-m PFS: 95.7%; 18-m PFS: 87.0%; 2-y PFS: 77.1%. |
| Rothschild/2021[16] | durvalumab+CT | 1-y OS: 91%(FAS); 2-y OS: 83%(FAS).  1-y EFS: 73.3%(FAS); 2-y EFS: 68%(FAS). |
| Shu/2020[18] | atezolizumab+CT | median DFS: 17.9m |
| Yang/2017[21] | ipilimumab+CT | median OS: 29.2m; 2-y OS: 73.0%. |

Abbreviations: ICI, checkpoint inhibitor; CT, chemotherapy; OS, overall survival; PFS, progression-free survival; DFS, disease-free survival; RFS, relapse-free survival; EFS, event-free survival; m, month; y, year.

**Table S4** Characteristics of studies of neoadjuvant chemotherapy

| First author/Year | Country | Phase  (design) | size | male  (%) | Median  age | SCC  (%) | Stage  1/2/3(%) | Chemotherapy  regimen | Type of resection(%)* |
| --- | --- | --- | --- | --- | --- | --- | --- | --- | --- |
| Forde/2021[13] | USA | III (dual-arm) | 179 | 71 | 65 | 53 | 4/31/65 | Platinum based | 61/25/13 |
| Lei/2020[14] | China | II (dual-arm) | 7 | NR | NR | NR | 0/0/100 | Paclitaxel+Cisplatin | NR |
| Date/2002[35] | Japan | I/II (single-arm) | 15 | 87 | 60 | 27 | 0/0/100 | Irinotecan+Cisplatin | NR |
| Marinis/2003[36] | Italy | II (single-arm) | 49 | 22 | 61 | 33 | 0/0/100 | Gemcitabine+Paclitaxel  +Cisplatin | 66/14/20 |
| Nagai/2003[37] | Japan | III (dual-arm) | 31 | 65 | 59 | 23 | 0/0/100 | Vindesine+Cisplatin | 75/7/18 |
| Brechot/2005[38] | France | II (dual-arm) | 19 | 83 | 54.9 | 43 | 0/0/100 | Gemcitabine+Cisplatin | NR |
|  |  |  | 11 | NR | NR | NR | 0/0/100 | Mltomycln+Ifosfamide  +Cisplatin | NR |
| Ramnath/2005[39] | USA | II (single-arm) | 62 | 73 | 65 | 48 | 44/24/32 | Gemcitabine+Vinorelbine | 69/24/7 |
| Abratt/2006[40] | South Africa | II (single-arm) | 44 | 89 | 56.4 | NR | 18/16/66 | Gemcitabine+Paclitaxel | NR |
|  |  |  |  |  |  |  |  | +Carboplatin |  |
| Betticher/2006[41] | Switzerland | II (single-arm) | 90 | 77 | 59 | 43 | 0/0/100 | Cisplatin+Docetaxel | 37/50/13 |
| Aydiner/2007[42] | Turkey | II (single-arm) | 47 | 98 | 56.3 | 47 | 48/26/26 | Gemcitabine+Cisplatin | NR |
| Gilligan/2007[43] | UK | dual-arm | 258 | 72 | 62 | 51 | 64/28/8 | Platinum based | 66/27/7 |
| Martins/2007[44] | USA | II (single-arm) | 30 | 66 | 56 | 34 | 30/23/47 | Platinum based | NR |
| Detterbeck/2008[45] | USA | II (triple-arm) | 87 | 53 | 63 | 39 | 70/30/0 | Gemcitabine based | 69/17/14 |
| Kunitoh/2008[46] | Japan | II (dual-arm) | 40 | NR | NR | 25 | 55/45/0 | Docetaxel+Cisplatin | 87/8/5 |
|  |  |  | 40 | NR | NR | 26 | 56/44/0 | Docetaxel | 86/3/11 |
| Thomas/2008[47] | Germany | dual-arm | 260 | 83 | 59 | 57 | 0/0/100 | Etoposide+Cisplatin | 51/35/13 |
| Girard/2009[48] | France | II (triple-arm) | 14 | 64 | 56 | 57 | 0/0/100 | Gemcitabine+Cisplatin | NR |
| LI/2009[49] | China | III (dual-arm) | 28 | 68 | 56 | 36 | 0/0/100 | Vinorelbine+Cisplatin | NR |
| Felip/2010[50] | Spain | III (triple-arm) | 199 | 88 | 65 | 54 | 75/23/2 | Paclitaxe+Carboplatin | NR |
| Pisters/2010[51] | USA | III (dual-arm) | 169 | 64 | 65 | 34 | NR | Paclitaxe+Carboplatin | 72/16/12 |
| Ahmed/2011[52] | USA | II (single-arm) | 20 | 40 | 65 | NR | 80/10/10 | Paclitaxel+Carboplatin | 83/17/0 |
| Berghmans/2012[53] | Belgium | II (dual-arm) | 69 | 52 | 64 | 40 | 40/22/38 | Mitomycin+Ifosfamide  +Cisplatin | 75/25 |
|  |  |  | 71 | 62 | 62 | 42 | 34/31/35 | Gemcitabine+Vinorelbine  +Cisplatin | 68/30/2 |
| Scagliotti/2012[54] | Italy | III (dual-arm) | 129 | 78 | 60.6 | 37 | 43/52/5 | Gemcitabine+Cisplatin | 72/17/11 |
| Chaft/2013[55] | USA | II (single-arm) | 50 | 40 | 61 | 0 | 12/20/68 | Cisplatin+Docetaxel  +Bevacizumab | NR |
| Dy/2014[56] | USA | II (single-arm) | 38 | 50 | 62.5 | 26 | 13/37/50 | Pemetrexed+Cisplatin | NR |
| Pless/2015[57] | Switzerland | III (dual-arm) | 115 | 67 | 59 | 31 | 0/0/100 | Docetaxel+Cisplatin | 63/20/17 |
| Cascone/2018[58] | USA | II (single-arm) | 47 | 62 | 64 | 38 | 32/28/40 | Cisplatin+Docetaxel | 84/11/5 |
| Hainsworth/2018[59] | USA | II (single-arm) | 46 | 39 | 65 | 0 | 11/37/52 | Pemetrexed+Carboplatin | 70/11/19 |
| Mittal/2021[60] | India | II (single-arm) | 37 | NR | 55 | 35 | 0/19/81 | Paclitaxel+Carboplatin | 82/18/0 |
| Zhu/2021[61] | China | II (single-arm) | 36 | 100 | 58 | 100 | 0/0/100 | Paclitaxel+Carboplatin | 70/17/13 |

Abbreviations: SCC, squamous cell carcinoma; NR, not reported. *: Type of resection (Lobectomy/Pneumonectomy/Others) (%).

**Table S5** Outcomes of studies of neoadjuvant chemotherapy

| First author/Year | Chemotherapy regimen | ORR | MPR rate | pCR rate | Surgical resection rate | R0 resection  rate | Incidence of surgical  complication | Incidence of grade 3-5 TRAEs |
| --- | --- | --- | --- | --- | --- | --- | --- | --- |
| Forde/2021[13] | Platinum based | 37% | 13% | 3% | 75% | 78% | 35% | 21% |
| Lei/2020[14] | Paclitaxel+Cisplatin | 57% | 33% | 17% | 86% |  | NR | NR |
| Date/2002[35] | Irinotecan+Cisplatin | 73% | NR |  | 100% | 73% | NR | NR |
| Marinis/2003[36] | Gemcitabine+Paclitaxel +Cisplatin | 73% | NR | 28% | 59% | 93% | NR | NR |
| Nagai/2003[37] | Vindesine+Cisplatin | 26% | NR | 0% | 74% | 87% | NR | NR |
| Brechot/2005[38] | Gemcitabine+Cisplatin | 58% | NR | 13% | 84% | 100% | NR | NR |
|  | Mltomycln+Ifosfamide +Cisplatin | 45% | NR | 11% | 82% | 100% | NR | NR |
| Ramnath/2005[39] | Gemcitabine+Vinorelbine | 34% | NR | 2% | 90% | 77% | NR | NR |
| Abratt/2006[40] | Gemcitabine+Paclitaxel +Carboplatin | 76% | NR | 12% | 93% | 88% | NR | NR |
| Betticher/2006[41] | Cisplatin+Docetaxel | 61% | NR | 19% | 83% | 57% | 17% | NR |
| Aydiner/2007[42] | Gemcitabine+Cisplatin | 57% | NR | 5% | 81% | 89% | NR | 55% |
| Gilligan/2007[43] | Platinum based | 47% | NR | 3% | 90% | 89% | NR | NR |
| Martins/2007[44] | Platinum based | 77% | NR | 18% | 73% | 95% | NR | NR |
| Detterbeck/2008[45] | Gemcitabine based | 29% | NR | 3% | 82% | 92% | NR | NR |
| Kunitoh/2008[46] | Docetaxel+Cisplatin | 45% | NR | 5% | 98% | 97% | 5% | NR |
|  | Docetaxel | 15% | NR | 0% | 88% | 97% | 0% | NR |
| Thomas/2008[47] | Etoposide+Cisplatin+ | 46% | 11% | NR | 59% | 55% | 12% | NR |
| Girard/2009[48] | Gemcitabine+Cisplatin | 57% | NR | 0% | 86% | 83% | NR | NR |
| LI/2009[49] | Vinorelbine+Cisplatin | 54% | NR | 8% | 89% | 88% | NR | NR |
| Felip/2010[50] | Paclitaxe+Carboplatin | 53% | NR | 10% | 91% | NR | NR | NR |
| Pisters/2010[51] | Paclitaxe+Carboplatin | 41% | NR | 10% | 90% | 93% | NR | NR |
| Ahmed/2011[52] | Paclitaxel+Carboplatin | 40% | NR | 17% | 90% | 100% | 10% | NR |
| Berghmans/2012[53] | Mitomycin+Ifosfamide +Cisplatin | 59% | NR | 14% | 86% | 90% | NR | NR |
|  | Gemcitabine+Vinorelbine +Cisplatin | 63% | NR | 2% | 85% | 95% | NR | NR |
| Scagliotti/2012[54] | Gemcitabine+Cisplatin | 34% | NR | 5% | 85% | 88% | 0% | 40% |
| Chaft/2013[55] | Cisplatin+Docetaxel +Bevacizumab | 40% | 27% | NR | 82% | 88% | 12% | NR |
| Dy/2014[56] | Pemetrexed+Cisplatin | 29% | NR | 0% | 89% | 94% | NR | NR |
| Pless/2015[57] | Docetaxel+Cisplatin | 43% | NR | 12% | 82% | 81% | NR | 60% |
| Cascone/2018[58] | Cisplatin+Docetaxel | 60% | 19% | 3% | 79% | 95% | 19% | NR |
| Hainsworth/2018[59] | Pemetrexed+Carboplatin | 41% | NR | 0% | 59% | 93% | NR | NR |
| Mittal/2021[60] | Paclitaxel+Carboplatin | 76% | 0% | 0% | 46% | 100% | NR | NR |
| Zhu/2021[61] | Paclitaxel+Carboplatin | 50% | 35% | 9% | 64% | 96% | NR | NR |

Abbreviations: ORR, objective response rate; MPR, major pathologic response; pCR, pathological complete response; TRAEs, treatment-related adverse events; NR, not reported.

**Table S6** Incidence of individual grade ≥3 TRAEs

| nICI combination, n(%) |  | nICI monotherapy, n(%) |
| --- | --- | --- |
| Total, 120(100) |  | Total, 21(100%) |
| Neutropenia, 30(25.0) |  | Pneumonia/Pneumonitis, 5(23.8) |
| AST/ALT increased, 12(10.0) |  | Hypokalemia, 3(14.3) |
| Pneumonia/Pneumonitis, 8(6.7) |  | Skin rash, 2(9.5) |
| Fatigue, 8(6.7) |  | Neutropenia, 1(4.8) |
| Thrombocytopenia, 6(5.0) |  | AST/ALT increased, 1(4.8) |
| Anemia, 6(5.0) |  | Renal insufficiency, 1(4.8) |
| Renal insufficiency, 6(5.0) |  | Fatigue, 1(4.8) |
| Lipase increased, 4(3.3) |  | Hyponatremia, 1(4.8) |
| Diarrhea, 4(3.3) |  | Blood creatine phosphokinase increased, 1(4.8) |
| Skin rash, 4(3.3) |  | Enterocolitis, 1(4.8) |
| Arthritis, 4(3.3) |  | Hypophysitis, 1(4.8) |
| Nausea, 3(2.5) |  | Arthritis, 1(4.8) |
| Hyperglycaemia, 3(2.5) |  | psoriasis flare, 1(4.8) |
| Hyperlipasaemia, 3(2.5) |  | Stroke, 1(4.8) |
| Serum amylase increased, 2(1.7) |  |  |
| Alopecia, 2(1.7) |  |  |
| Neurotoxicity, 2(1.7) |  |  |
| Pruritus, 2(1.7) |  |  |
| Cardiopulmonary event, 2(1.7) |  |  |
| Weight loss, 1(0.8) |  |  |
| Constipation, 1(0.8) |  |  |
| Hyponatremia, 1(0.8) |  |  |
| pulmonary embolism, 1(0.8) |  |  |
| seizure, 1(0.8) |  |  |
| bronchopleural fistula, 1(0.8) |  |  |
| Hypophysitis, 1(0.8) |  |  |
| Autoimmune hepatitis, 1(0.8) |  |  |
| ARDS, 1(0.8) |  |  |

Abbreviations: nICI, neoadjuvant checkpoint inhibitor; TRAEs, treatment-related adverse events.

**Table S7** Characteristics and main findings of the previous meta-analyses

| First author /Published year | Design of included studies | No. of studies of nICI included | Stage | No. of patients | Main finding | Subgroup analysis |
| --- | --- | --- | --- | --- | --- | --- |
| Xue/2022[68] | Phase 1-2 | 21 | 1a-3b | 792 | Operation rate and R0 resection rate were 92% and 97%. MPR and pCR rates were 39% and 25%. Rate of any-grade and grade 3–5 TRAEs were 57% and 15%. | area, arms, nICI modes and ICI types |
| Jiang/2022[69] | Prospective | 15 | 1-3 | 809 | MPR rate was 43.5% and pCR rate was 21.9%. Resection rate was 85.8% and the surgical delay rate was 7.4%. nICI+CT had improved MPR than nICI monotherapy. Patients with PD-L1≥1% had better pathological responses. | nICI modes, ICI types, PD-L1 expression, histology, and smoking |
| Deng/2022[70] | Phase 1-3 | 10 | 1a-3b | 461 | Compared with PD-L1 expression <1%, PD-L1 expression ≥1% is associated with a higher rate of MPR (OR=2.62, P<0.001) and pCR (OR=2.94, P<0.001). | nICI modes |
| Cao/2021[71] | Prospective | 16 | 1a-3b | 548 | MPR and pCR rates were 52% and 20%. Mortality within 30 days was 0.6%. | none |
| Zhang/2021[72] | Phase 1-2 | 10 | 1-3 | 400 | nICI+CT (36.2%) showed an increased pCR rate compared with nICI alone (10.6%) or nCT (7.5%). nCT (87.2%) showed a lower R0 resection rate compared with nICI alone (92.7%) or nICI+CT (91.6%). | none |
| Jia/2020[73] | phase 1-3 | 7 | 1-3a | 252 | Compared with nCT, nICI showed significantly higher MPR and pCR rates (MPR: OR = 0.59; pCR: OR = 0.16). The pooled OR values of the incidence of TRAE, surgical complications and surgical delay rate were 0.19, 0.41 and 0.03, respectively, which were significantly better than those for nCT. | none |

Abbreviations: nICI, neoadjuvant immune checkpoint inhibitor; nCT, neoadjuvant chemotherapy; ORR, objective response rate; MPR, major pathologic response; pCR, pathological complete response; TRAEs, treatment-related adverse events.


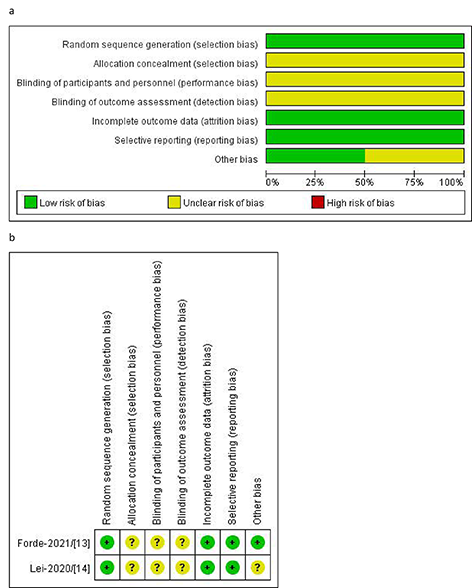


**Figure S1** Assessment of risk of bias. a: Methodological quality graph: authors’ judgment about each methodological quality item presented as percentages across all included studies; b: Methodological quality summary: authors’ judgment about each methodological quality item for each included study, “+” low risk of bias; “?” unclear risk of bias; “-” high risk of bias.


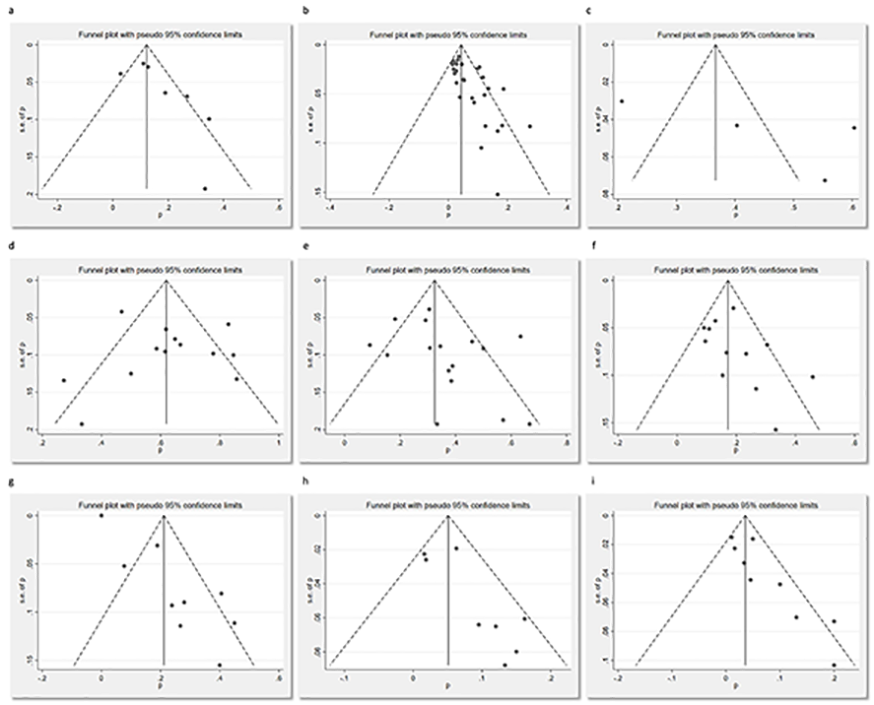


**Figure S2** Funnel plots of publication bias. a, MPR for neoadjuvant CT; b, pCR for neoadjuvant CT; c, grade ≥3 TRAEs for neoadjuvant CT; d, MPR for ICI combination; e, pCR for ICI combination; f, grade ≥3 TRAEs for ICI combination; g, MPR for ICI monotherapy; h, pCR for ICI monotherapy; i, grade ≥3 TRAEs for ICI monotherapy. MPR, major pathologic response; pCR, pathological complete response; TRAEs, treatment-related adverse events; CT, chemotherapy; ICI, checkpoint inhibitor.


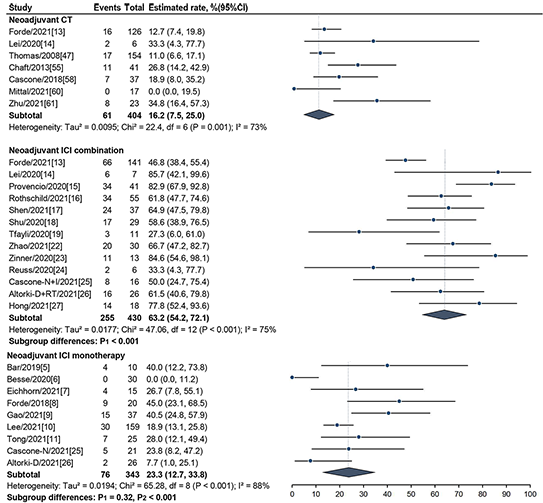


**Figure S3** Forest plot of MPR rate. MPR, major pathologic response; ICI, checkpoint inhibitor; CT, chemotherapy.


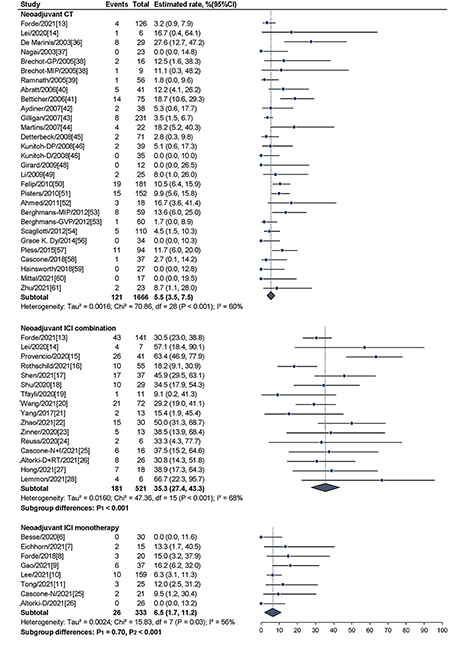


**Figure S4** Forest plot of pCR rate. pCR, pathological complete response; ICI, checkpoint inhibitor; CT, chemotherapy.


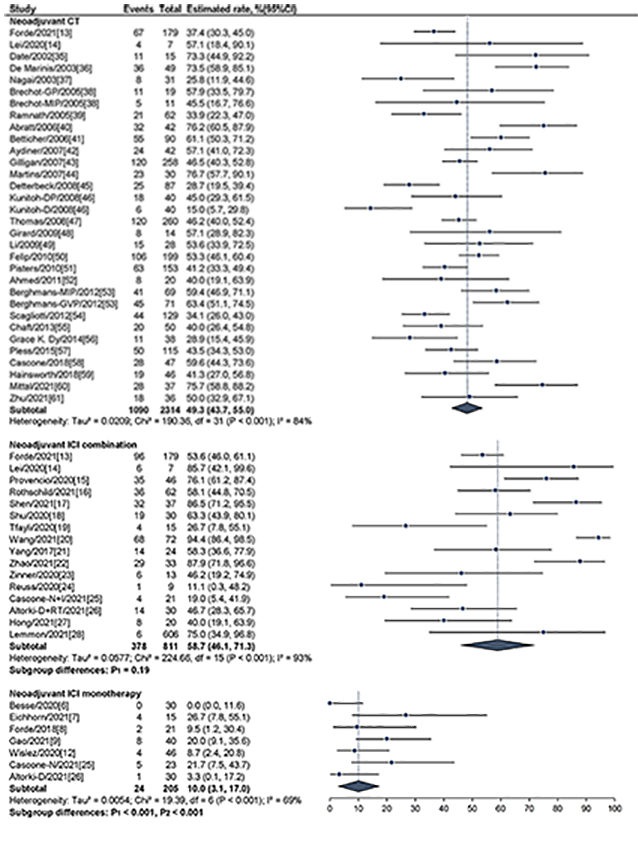


**Figure S5** Forest plot of ORR. ORR, objective response rate; ICI, checkpoint inhibitor; CT, chemotherapy.


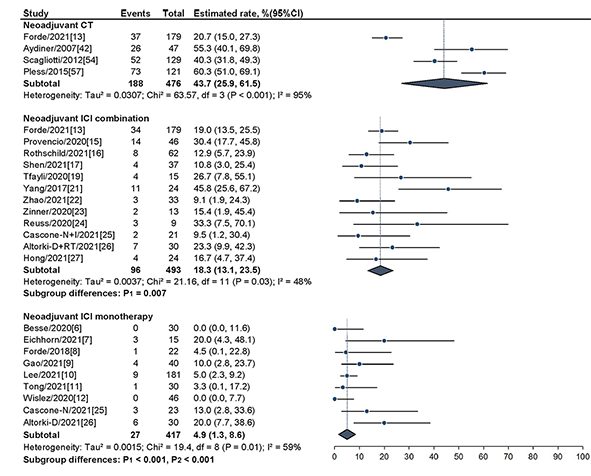


**Figure S6** Forest plot of incidence of grade ≥3 TRAEs. TRAEs, treatment-related adverse events; ICI, checkpoint inhibitor; CT, chemotherapy.

**
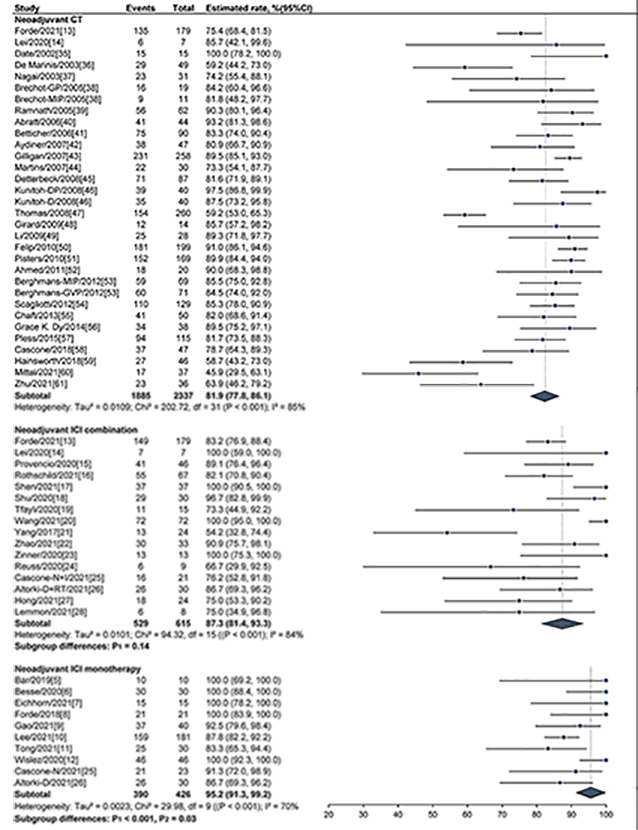
**

**Figure S7** Forest plot of surgical resection rate. ICI, checkpoint inhibitor; CT, chemotherapy.

**
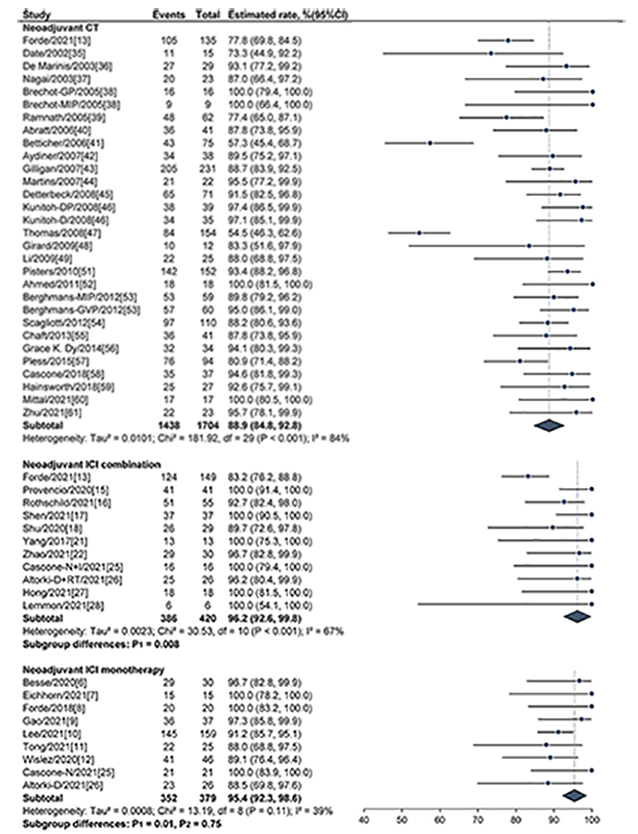
**

**Figure S8** Forest plot of surgical R0 resection rate. ICI, checkpoint inhibitor; CT, chemotherapy.

**
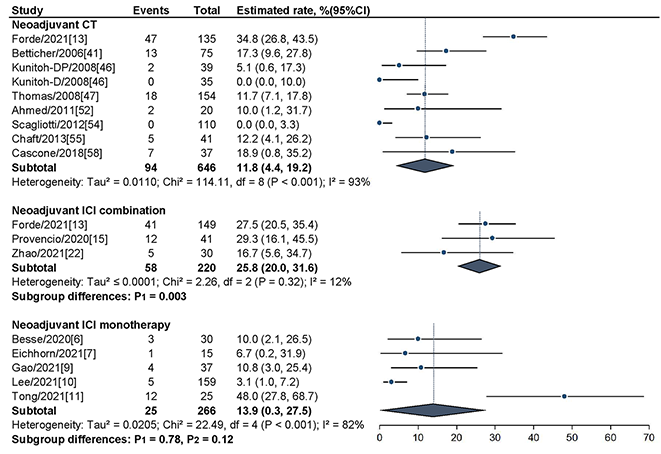
**

**Figure S9** Forest plot of incidence of surgical complication. ICI, checkpoint inhibitor; CT, chemotherapy.

**
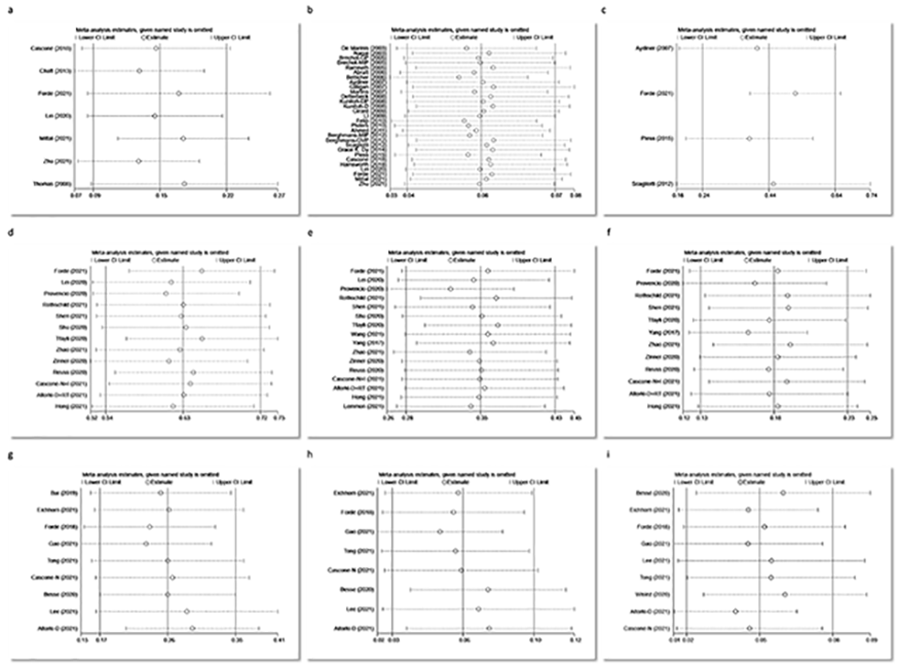
**

**Figure S10** Sensitivity analysis. a, MPR for neoadjuvant CT; b, pCR for neoadjuvant CT; c, grade ≥3 TRAEs for neoadjuvant CT; d, MPR for ICI combination; e, pCR for ICI combination; f, grade ≥3 TRAEs for ICI combination; g, MPR for ICI monotherapy; h, pCR for ICI monotherapy; i, grade ≥3 TRAEs for ICI monotherapy. CT, chemotherapy; ICI, checkpoint inhibitor; MPR, major pathologic response; pCR, pathological complete response; TRAEs, treatment-related adverse events; ICI, checkpoint inhibitor.

**
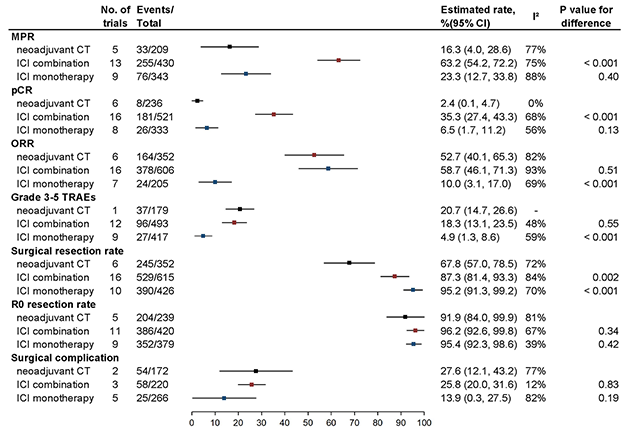
**

**Figure S11** Outcomes of neoadjuvant ICI vs neoadjuvant chemotherapy in studies published within the last 5 years (since 2017). MPR, major pathologic response; pCR, pathological complete response; ORR, objective response rate; TRAEs, treatment-related adverse events; ICI, checkpoint inhibitor; CT, chemotherapy.

**
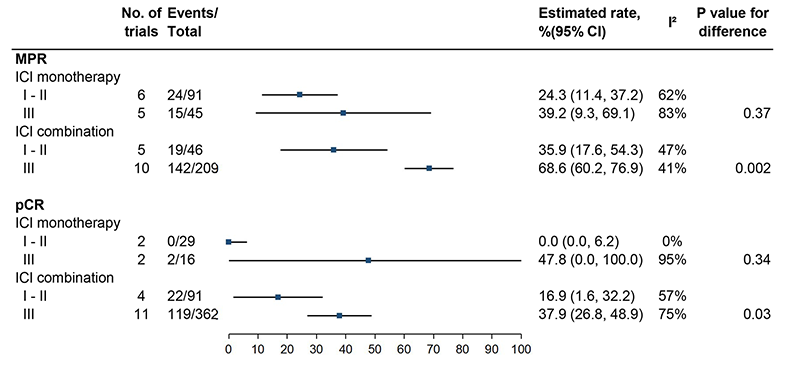
**

**Figure S12** MPR and pCR rates of patients with stage I-II vs stage III according to treatment mode. MPR, major pathologic response; pCR, pathological complete response; ICI, checkpoint inhibitor.


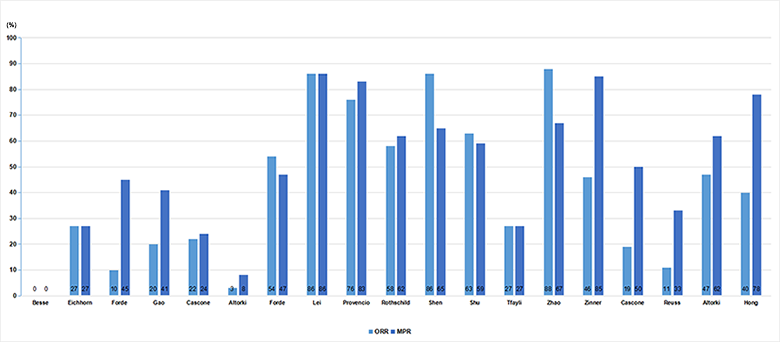


**Figure S13** MPR rate and ORR in individual studies of neoadjuvant ICI. ORR, objective response rate; MPR, major pathologic response; ICI, checkpoint inhibitor.
